# The Microalgal Diatoxanthin Inflects the Cytokine Storm in SARS-CoV-2 Stimulated ACE2 Overexpressing Lung Cells

**DOI:** 10.3390/antiox11081515

**Published:** 2022-08-03

**Authors:** Clementina Sansone, Luigi Pistelli, Angelo Del Mondo, Luana Calabrone, Angelo Fontana, Douglas M. Noonan, Adriana Albini, Christophe Brunet

**Affiliations:** 1 Stazione Zoologica Anton Dohrn, sede Molosiglio Marina Acton, Via Ammiraglio F. Acton 55, 80133 Napoli, Italy; luigi.pistelli@szn.it (L.P.); angelo.delmondo@szn.it (A.D.M.); luana.calabrone@szn.it (L.C.); 2 Institute of Biomolecular Chemistry, CNR, Via Campi Flegrei 34, Pozzuoli, 80078 Napoli, Italy; angelo.fontana@icb.cnr.it; 3 Unit of Molecular Pathology, Biochemistry and Immunology, IRCCS MultiMedica, 20138 Milan, Italy; douglas.noonan@uninsubria.it; 4 Department of Biology, University of Naples “Federico II”, Via Cupa Nuova Cinthia 21, 80126 Napoli, Italy; 5 Department of Biotechnology and Life Sciences, University of Insubria, 21100 Varese, Italy; 6 IRCCS European Institute of Oncology, IEO, 20141 Milan, Italy; adriana.albini@ieo.it

**Keywords:** carotenoids, ACE2, COVID-19, inflammation, cytokine, antiviral, diatoms

## Abstract

Contact between SARS-CoV-2 and human lung cells involves the viral spike protein and the human angiotensin-converting enzyme 2 (ACE2) receptor on epithelial cells, the latter being strongly involved in the regulation of inflammation as well as blood pressure homeostasis. SARS-CoV-2 infection is characterized by a strong inflammatory response defined as a “cytokine storm”. Among recent therapeutic approaches against SARS-CoV-2 targeting the dramatic inflammatory reaction, some natural products are promising. Diatoms are microalgae able to produce bioactive secondary metabolites, such as the xanthophyll diatoxanthin (Dt). The aim of this study is to demonstrate the anti-inflammatory effects of Dt on the A549-hACE2 lung cell line, exploring its interaction with the ACE2 receptor, as well as depicting its role in inhibiting a cytokine storm induced by the SARS-CoV-2 spike glycoprotein. Results showed that Dt enhanced the cell metabolism, e.g., the percent of metabolically active cells, as well as the ACE2 enzymatic activity. Moreover, Dt strongly affected the response of the SARS-CoV-2 spike glycoprotein-exposed A549-hACE2 cells in decreasing the interleukin-6 production and increasing the interleukin-10 release. Moreover, Dt upregulated genes encoding for the interferon pathway related to antiviral defense and enhanced proteins belonging to the innate immunity response. The potential interest of Dt as a new therapeutic agent in the treatment and/or prevention of the severe inflammatory syndrome related to SARS-CoV-2 infection is postulated.

## 1. Introduction

The first interaction between SARS-CoV-2 and human lung cells corresponds to the recognition of the viral spike protein by the human angiotensin-converting enzyme 2 (ACE2) receptor on epithelial cells in the airways. This is followed by virus entry and priming of human transmembrane protease serine 2 (TMPRSS2), which cleaves the S protein and initiates viral fusion [1]. One of the most pronounced cellular responses to SARS-CoV-2 in lung cells is the cytokine storm [2], which can dramatically lead to acute respiratory distress syndrome (ARDS), with subsequent lung injury [3]. This storm results from the sudden acute increase in circulating levels of different pro-inflammatory cytokines, including the interleukins IL-6, IL-1, and the TNFα that are the most important pro-inflammatory mediators of the innate immune response [4,5,6]. This detrimental biological event is therefore a target for a therapeutic solution to SARS-CoV-2 infection; for instance, the tocilizumab drug that acts on the cytokine IL-6 [7]. Anti-inflammatory therapies fighting the cytokine responses are suggested to decrease both the morbidity and mortality in infected SARS-CoV-2 patients [8], such as nonsteroidal anti-inflammatory drugs [9]. Natural compounds/extracts, e.g., from the vegetal kingdom, exhibiting antimicrobial or antiviral properties are strongly attractive [10]. Natural compounds with anti-inflammatory activities are also appealing [11]; for instance, those displaying interests against SARS-CoV-2 [12,13,14,15,16,17]. Although some terrestrial plants are currently used as human health benefit resources [18], there is a growing interest in marine microalgae [19], thanks to the scientifically recognized strengths offered by those organisms [20,21]. Among microalgae, diverse families of bioactive compounds show antiviral properties, as reported for sulfated polysaccharides [22] or polyphenols [23]. Moreover, carotenoids, among the most bioactive compound families, do present some antiviral potential, such as the exclusively aquatic species, astaxanthin [24], fucoxanthin, or siphonaxanthin [25]. Nevertheless, in silico analysis carried out on nine xanthophylls demonstrated their ability to inhibit the main protease (Mpro) and the papain-like protease (PLpro) of SARS-CoV-2 [26]. It is noteworthy that microalgae present a high diversity of carotenoids, with some of them understudied [27]. This is the case of the xanthophyll diatoxanthin (Dt), belonging to diatoms and exerting a (photo-)protective role [28,29,30,31,32]. Recently, its great antioxidant property and in vitro bioactivity have been demonstrated ([27], unpublished data), satisfying chemopreventive and anti-inflammatory potential, e.g., inhibiting a cytokine storm [27,33]. These recent studies pave the way for further investigation on the human health-induced bioactivity of Dt. The aim of the present study is to underline in vitro the diatoxanthin effects on the expressing ACE2 carcinoma cell line, A549-hACE2. On the one hand, it focused on the interactions between Dt and the ACE2 receptor, performing a docking analysis, and on the ACE2 enzymatic activity. On the other hand, the study explores the Dt role in inhibiting a cytokine storm induced by the SARS-CoV-2 spike glycoprotein, investigating at the molecular and protein levels. The potential interest of using Dt as a new therapeutic agent in the treatment and/or prevention of the severe inflammatory syndrome related to SARS-CoV-2 infection is discussed.

## 2. Materials and Methods

### 2.1. The A549-hACE2 Cell Line Cultivation

The lung carcinoma cell line (A549-hACE2) was purchased from Invivogen (cat. a549-hace2, Invivogen, San Diego, CA, USA). The A549-hACE2 cells, generated from the A549 lung carcinoma cell line, were stably transfected to express the human ACE2 (hACE2) gene. The A549-hACE2 cell line is an *in vitro* model commonly used for SARS-CoV-2 investigations [34,35].

The A549-hACE2 cell line was grown in cultured DMEM/F12 (Dulbecco’s odified Eagle’s medium/nutrient mixture F-12 Ham), supplemented with 10% FBS, 100 units mL^−1^ penicillin and 100 units mL^−1^ streptomycin, in a 5% CO_2_ atmosphere at 37 °C. Cells were cultured until reaching an adequate confluence, and then seeded in multi-well plates for the experimental purposes described below.

### 2.2. Diatoxanthin

Pure diatoxanthin (C_40_H_54_O_2_, CAS No. 31063-73-7; Figure S1) was purchased from D.H.I. Water & Environment (Hørsholm, Denmark). The purity was then assessed through HPLC analysis, using the method described in Pistelli et al. [36].

### 2.3. MTT (3-(4,5-Dimethylthiazol-2-yl)-2,5-diphenyltetrazolium Bromide) Assay

The A549-hACE2 cells (2 × 10^3^ cells well^−1^, 100 μL well^−1^) were seeded in 96-well plates (TPP Techno Plastic Products AG, Trasadingen, Switzerland) and kept overnight for attachment. Cells were incubated for 48 h with Dt in DMSO at four different concentrations, namely 0.1, 1, 10, and 100 ng mL^−1^. The final concentration of DMSO was 0.1% *v*/*v* for the highest concentration of Dt (100 ng). At the end of the 48 h-incubation, Orangu^TM^ (Cell Guidance Systems, Cambridge, UK) was used to measure cell viability according to the manufacturer’s instructions. The absorbance was recorded using a Microplate Reader: Infinite^®^ M1000 PRO (TECAN, Männedorf, Switzerland) at a wavelength of 450 nm. The same analysis was conducted incubating cells with DMSO without Dt (untreated cells = control). The results were represented as a percent of metabolically active cells estimated as the ratio between the absorbance of each sample (A549-hACE2 cells treated with Dt) and the absorbance of the control (untreated cells).

### 2.4. Angiotensin-Converting Enzyme 2 (ACE2) Activity Assay

The A549-hACE2 cells (2 × 10^6^ cells well^−1^, 2 mL well^−1^) were seeded in 6-well plates (TPP Techno Plastic Products AG, Trasadingen, Switzerland) and kept overnight for attachment. Cells were then incubated with Dt in DMSO at the concentration 0.1 ng mL^−1^. Control was setup with A549-hACE2 cells plus DMSO without Dt addition. After 24 h incubation, the medium was removed from each well and the attached cells were rapidly washed with phosphate buffered saline (PBS) solution. The PBS solution was then removed, and the cell lysates were prepared by scraping each well into 500 μL of RIPA Lysis and Extraction Buffer (Thermo Fisher Scientific, Waltham, MA, USA), supplemented with Halt™ Protease & Phosphatase Inhibitor Cocktail (Thermo Fisher Scientific, Waltham, MA, USA). The lysates were incubated on ice for 5 min and then clarified by centrifugation at 14,000× *g* for 5 min. Total protein concentration was determined using a NanoDrop 1000 UV-Vis Spectrophotometer (Thermo Fisher Scientific, Waltham, MA, USA) at 280 nm. Reading at 260 nm was done to assess the purity of extracted proteins. ACE2 activity was measured using Angiotensin II Converting Enzyme (ACE2) Inhibitor Screening Kit (cat. No. MAK378, Sigma-Aldrich, St. Louis, MO, USA). The assay was performed in triplicate, using an aliquot of 10 μL of each sample and following the manufacturer’s protocol. Fluorescence was read using a Microplate Reader: Infinite^®^ M1000 PRO (Ex: 320 nm, Em: 420 nm, TECAN, Männedorf, Switzerland).

### 2.5. Target Fishing and Docking Analysis

The Dt molecule interaction with the ACE2 protein was retrieved through an introductory target fishing approach by using the ACID online server [37]. The 3D coordinates of the crystal structure of ACE2 were retrieved from the Protein Data Bank (PDB; PDB id: 1R42). The protein structure was optimized by using UCSF Chimera 1.16 software [38] for removal of all heteroatoms and water molecules, while further polar hydrogen atoms were added to protein to make the receptor molecules suitable for docking. Diatoxanthin (CID 6440986) was prepared as a ligand for docking, following the same procedure. Gasteiger charges were added and the maximum six numbers of active torsions were given to the lead compounds. ACE2 protein was finally docked using the molecular docking program AutoDock Vina 4.2.6 (Scripps Research, San Diego, CA, USA) [39].

### 2.6. Interleukins IL-6 and IL-10 Concentrations through Enzyme-Linked Immunosorbent Assay (ELISA)

The A549-hACE2 cells (2 × 10^6^ cells wells^−1^, 2 mL well^−1^) were seeded in 6-well plates (TPP Techno Plastic Products AG, Trasadingen, Switzerland) and kept overnight for attachment. Cells were incubated with Dt in DMSO at the concentration of 0.1, 1, 10, and 100 ng mL^−1^, with or without recombinant human coronavirus SARS-CoV-2 spike glycoprotein RBD (His tag) (cat. No. ab275986, Abcam, Darmstadt, Germany). After the 24 h-lasting incubation, cell media were collected and the evaluation of the interleukins IL-6 and IL-10 concentrations was carried out through sandwich ELISA. Human IL-6 Standard ABTS ELISA Development Kit (cat. No. 900-K16, PeproTech, London, UK) and Human IL-10 Standard ABTS ELISA Development Kit (cat. No. 900-K21, PeproTech, London, UK) were used according to the manufacturer’s protocol. The absorbance was measured at 405 nm (with wavelength correction set at 650 nm) using the Microplate Reader: Infinite^®^ M1000 PRO (TECAN, Männedorf, Switzerland).

### 2.7. RNA Extraction and PCR Array Gene Expression Analysis

The A549-hACE2 cells (2 × 10^6^ cells wells^−1^, 2 mL well^−1^) were seeded in 6-well plates (TPP Techno Plastic Products AG, Trasadingen, Switzerland) and kept overnight for attachment. Cells were treated for 2 h with Dt in DMSO at a concentration of 0.1 ng mL^−1^, while the control condition consisted of A549-hACE2 cells incubated with DMSO without Dt. At the end of the incubation, the medium was removed, and the attached cells were rapidly washed with cold phosphate buffered saline (PBS) solution and lysed directly in plates by adding 500 μL of TRIsure™ reagent (cat. No. BIO-3803, Meridian Bioscience Inc., Cincinnati, OH, USA). RNA was then isolated according to manufacturer’s protocol. RNA concentration and purity were assessed using the NanoDrop 1000 UV-Vis Spectrophotometer (Thermo Fisher Scientific, Waltham, MA, USA). The reverse transcription reaction was carried out using RT^2^ First Strand Kit (cat. No. 330404, Qiagen, Hilden, Germany). Real-time quantitative polymerase chain reaction (RT-qPCR) was performed using RT^2^ Profiler PCR Array Human Antiviral Response (384-well format, cat. No. 330231, PAHS-122ZA, Qiagen, Hilden, Germany). Plates were run on a ViiA 7 Real-Time PCR System (Thermo Fisher Scientific, Waltham, MA, USA). Standard fast PCR cycling protocol was run with 10 µL reaction volumes. Cycling conditions were set up in three stages: the first stage at 50 °C for 2 min and 95 °C for 10 min; the second stage consisted of 40 cycles at 95 °C for 15 s and 60 °C for 1 min; the last stage (melt curve) at 95 °C for 15 s, 60 °C for 1 min, and 95 °C for 15 s. qPCR data (Ct-values) were analyzed with PCR Array Data Analysis Online Software (Qiagen, Hilden, Germany). Relative gene expression values greater or lower than 2.0-expression ratio indicated significant gene expression variations with respect to the control. Genes used as control were actin-beta (ACTB), beta-2-microglobulin (B2M), glyceraldehyde-3 phosphate dehydrogenase (GAPDH), hypoxanthine phosphoribosyl transferase 1 (HPRT1), and ribosomal protein large P0 (RPLP0).

### 2.8. Tumor Necrosis Factor Receptor 1 (TNFR1) and Receptor Interacting Protein 2 (RIP2) Concentration

The production of tumor necrosis factor receptor 1 (TNFR1) and receptor interacting protein 2 (RIP2) was assessed through Western blot analysis. The A549-hACE2 cells (2 × 10^6^ cells wells^−1^, 1 mL well^−1^) were seeded in 12-well plates (TPP Techno Plastic Products AG, Trasadingen, Switzerland) and kept overnight for attachment. A group of A549-hACE2 cells was treated with interleukin 1β (IL-1β) at a concentration of 40 pg mL^−1^ for 24 h. After this time, Dt was added at a concentration of 0.1 ng mL^−1^ in half of the plates and incubated for 6 h, whereas in the other half of the plates, DMSO was added. Another group of A549-hACE2 cells was incubated for 24 h without IL-1β and then treated with Dt in DMSO at a concentration of 0.1 ng mL^−1^ for 6 h, while another with DMSO without Dt for 6 h. The medium was then removed, and the cell lysates were prepared by scraping each well into 500 μL of RIPA Lysis and Extraction Buffer (Thermo Fisher Scientific, Waltham, MA, USA), supplemented with Halt™ Protease and Phosphatase Inhibitor Cocktail (Thermo Fisher Scientific, Waltham, MA, USA). The lysates were incubated on ice for 5 min and then clarified by centrifugation at 14,000× *g* for 5 min. Total protein concentration was determined using a NanoDrop 1000 UV-Vis Spectrophotometer (Thermo Fisher Scientific, Waltham, MA, USA) at 280 nm. Reading at 260 nm was done to assess the purity of extracted proteins. Protein samples were incubated at 95 °C for 5 min before the separation of total polypeptides by SDS-PAGE electrophoresis, using 4–15% Criterion™ TGX Stain-Free™ Protein Gel (cat. No. 5678085, Bio-Rad, Hercules, CA, USA). The SDS-PAGEs were then blotted onto Trans-Blot Turbo Midi 0.2 μm Nitrocellulose membrane (cat. No. 170-4159, Bio-Rad, Hercules, CA, USA) using a Trans-Blot Turbo Transfer System (cat. No. 170-4150, Bio-Rad, Hercules, CA, USA). Membranes were incubated for 1 h in blocking reagent (1X Tris Buffered Saline-TBS), 0.1% Tween-20 with 5% w/v nonfat dry milk and incubated overnight at 4 °C with the primary antibodies diluted in 1X TBS, 0.1% Tween-20 with 5% BSA. TNFR1 antibody (1:1000 diluted, cat. No. 3736, Cell Signaling Technology Inc., Danvers, MA, USA) and RIP2 antibody (1:1000 diluted, cat. No. 4142, Cell Signaling Technology Inc., Danvers, MA, USA) were added and incubated overnight at 4 °C. After incubation, membranes were washed three times for 10 min each with 15 mL of TBS/Tween and then incubated with goat anti-rabbit IgG F(ab’)2 Secondary Antibody, HRP (1:500 dilution, cat. No. 31461, Invitrogen, Waltham, MA, USA) with gentle agitation for 2 h at room temperature. After incubation, membranes were washed three times for 10 min each with 15 mL of TBS/Tween. Blotted membranes were detected by using Clarity Western ECL Substrate (cat. No. 1705061, Bio-Rad, Hercules, CA, USA) and ChemiDoc™ MP Imaging System (cat. No. 120-3154, Bio-Rad, Hercules, CA, USA). Densitometric analysis of five immune-positive bands was performed using ImageLab software (Bio-Rad, Hercules, CA, USA).

### 2.9. Cytokine Release from A549-hACE2 Cells

The release of 120 cytokines in the media from the A549-hACE2 cells was analyzed through an antibody array using RayBiotech^®^ C-Series Human Cytokine Array C1000 (cat. No. AAH-CYT-1000, RayBiotech, Peachtree Corners, GA, USA). The experimental procedure was the same as previously described for the Western blot analysis. The A549-hACE2 cells (2 × 10^6^ cells wells^−1^, 1 mL well^−1^) were seeded in 12-well plates (TPP Techno Plastic Products AG, Trasadingen, Switzerland) and kept overnight for attachment. A group of A549-hACE2 cells was treated with interleukin 1β (IL-1β) at a concentration of 40 pg mL^−1^ for 24 h in order to simulate the virus intracellular entry effect, when patients have high fever and headache as initial symptoms. After this time, Dt was added at a concentration of 0.1 ng mL^−1^ in half of the plates and incubated for 6 h, whereas in the other half of the plates, DMSO was added. Another group of A549-hACE2 cells was incubated for 24 h without IL-1β and then treated with Dt in DMSO at a concentration of 0.1 ng mL^−1^ for 6 h, while another with DMSO without Dt for 6 h. After incubation, cell medium was collected and protein concentration and purity were assessed at 280 and 260 nm, respectively, using a NanoDrop 1000 UV-Vis Spectrophotometer (Thermo Fisher Scientific, Waltham, MA, USA). For each condition tested, 1 mL of sample was used to perform the antibody array, according to the manufacturer’s protocol. Blots were analyzed using ImageLab software (Bio-Rad, Hercules, CA, USA). Results are represented as the ratio between the expression of cytokines in the medium of A549-hACE2 cells incubated with IL-1β and recovered with Dt vs. cells only incubated with IL-1β.

## 3. Results

### 3.1. Dt Effect on the A549-hACE2 Cell Line Viability and ACE2 Activity

**Figure 1:**
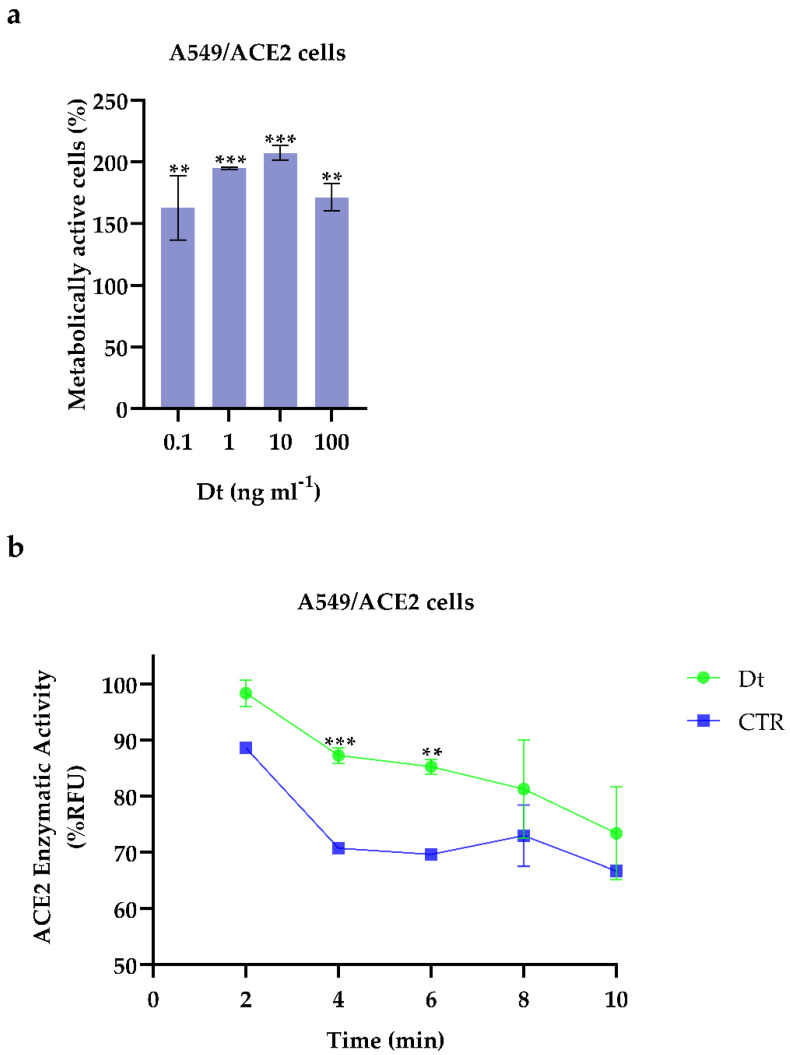
(**a**) Cell viability assays on A549-hACE2 cells treated for 48 h at four Dt concentrations. Values are expressed as mean ± SD of metabolically active cells compared to control (no Dt, 100% of metabolically active cells). All concentrations tested showed significant differences compared to the control (no Dt, *p*-value ≤ 0.0001; Dunnet’s test) (**b**) ACE2 enzymatic activity screening assay on A549-hACE2 cells treated for 24 h with 0.1 ng mL^−1^ of Dt. Values are expressed as mean ± SD of %RFU (Relative Fluorescence Units). Asterisks indicate the statistically significant difference compared to the respective control *** *p* ≤ 0.001, ** *p* ≤ 0.01; Sidak’s test).

Dt did not exert any cytotoxicity on A549-hACE2 cells (Figure 1a). Conversely, it did significantly enhance the metabolic activity of the A549-hACE2 cells, already at the lowest concentration (0.1 ng mL^−1^) compared to the control (at least *p* ≤ 0.01; Figure 1a). From this first result, we selected the lowest concentration of Dt, 0.1 ng mL^−1^, to further evaluate the role and effects of Dt on counteracting inflammation processes in A549-hACE2 cells.

### 3.2. Angiotensin Converting Enzyme 2 (ACE2) Activity and Molecular Docking between Dt and ACE2

**Figure 2:**
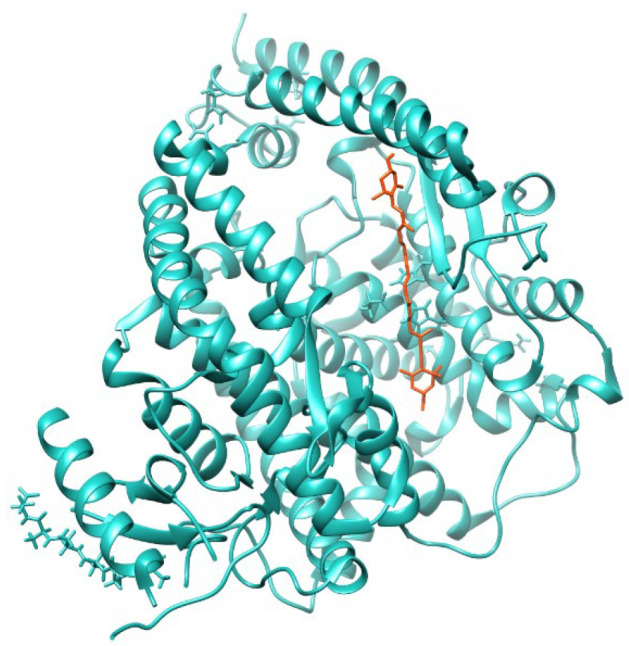
Molecular docking diagrams of diatoxanthin binding to ACE2.

In the presence of Dt, the angiotensin converting enzyme 2 (ACE2) enzymatic activity in A549-hACE2 cells significantly increased (*p* ≤ 0.01) compared to the control (Figure 1b). Docking analysis revealed that diatoxanthin binds with the ACE2 (Figure 2), a chemical interaction that confirmed the effects of Dt on ACE2. This is due to hydrophobic interaction (ΔE = −7.9 kcal mol^−1^) established within the alpha-helix in the N domain involving the cleavage site of the enzyme. Docked conformation over-imposes standard ligand conformation (RMSD < 1).

### 3.3. Interleukin (IL-6 and IL-10) Production

**Figure 3:**
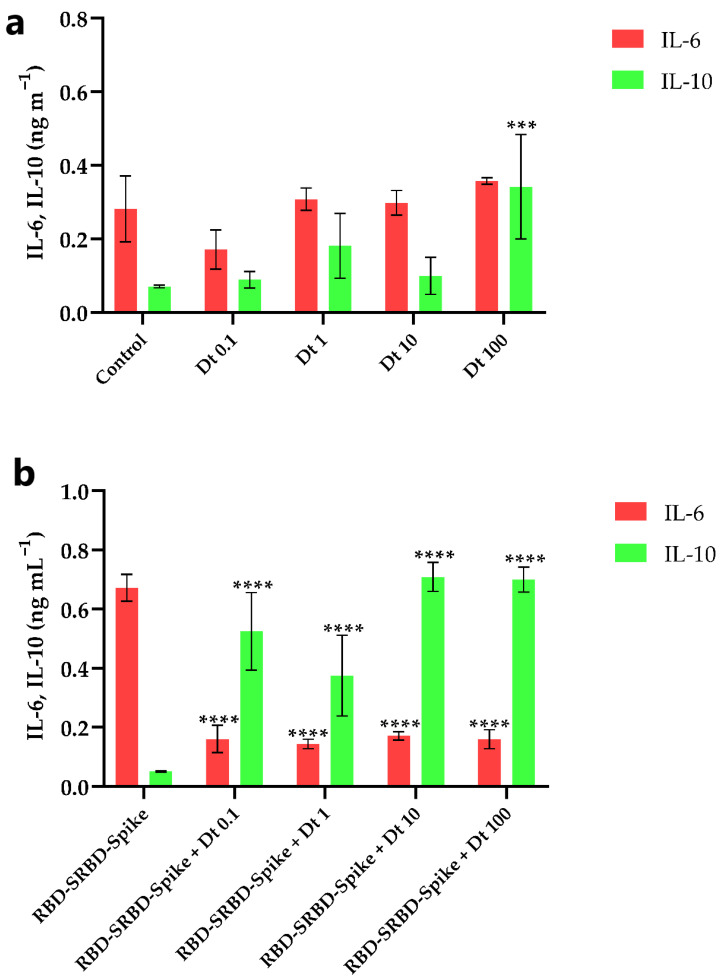
Extracellular IL-6 and IL-10 concentration in A549-hACE2 cell cultivation treated with four concentrations of Dt, in the absence (**a**) and presence (**b**) of recombinant human coronavirus SARS-CoV-2 spike glycoprotein RBD (RBD-SRBD-Spike). Values are expressed as mean ± SD of IL-6 or IL-10 (ng mL^−1^). Asterisks indicate the statistically significant difference compared to the respective control, namely (**a**) no Dt in the absence of RBD-SRBD-Spike, and (**b**) no Dt in the presence of RBD-SRBD-Spike (**** *p* ≤ 0.0001, *** *p* ≤ 0.001; Dunnet’s test).

The Dt mediating effect on both IL-6 and IL-10 release was targeted in the A549-hACE2 cells in the absence or presence of SARS-CoV-2 spike glycoprotein. IL-6 and IL-10 have a role on the cytokine storm occurring in the inflamed cells infected by SARS-CoV-2 [6], and as an anti-inflammatory response affected by SARS-CoV-2 [5,40], respectively. In the absence of SARS-CoV-2 spike glycoprotein, Dt did not significantly affect IL-6 release compared to the control (*p* > 0.05; Figure 3a), whereas it did significantly increase the IL-10 production when applied at the highest concentration (100 ng mL^−1^, *p* < 0.001; Figure 3a).

The contact between SARS-CoV-2 spike glycoprotein and A549-hACE2 cells strongly enhanced the extracellular IL-6 release and significantly lowered the IL-10 release (*p* < 0.001; Figure 3a,b). In this inflammatory context, Dt decreased the IL-6 production (*p* < 0.0001; Figure 3b) and induced an increase of the IL-10 production (at least *p* < 0.05; Figure 3b).

### 3.4. Dt-Modulated Gene Expression in A549-hACE2 Cells

**Figure 4:**
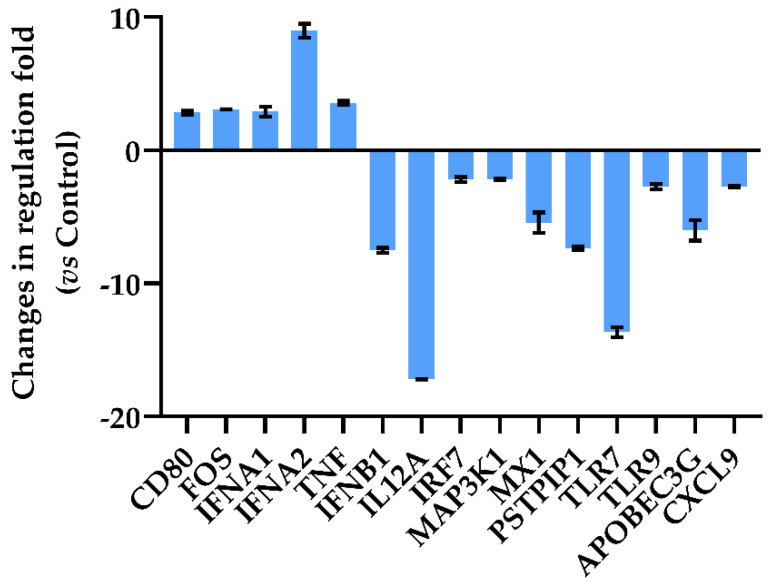
Quantitative RT-PCR of oxidative stress-related genes in A549-hACE2 cells after 2 h of treatment with 0.1 ng mL^−1^ of Dt. Values are expressed as mean ± SD of changes in fold-regulation compared to the control (no Dt). All data from gene expression analysis are included in Table S1.

Over the selected 84 genes involved in antiviral innate immunity cell response (Table S1), the A549-hACE2 cells treated with Dt at 0.1 ng mL^−1^ significantly modulated 15 genes related to the inhibition of the inflammatory response by decreasing all trigger factors in antiviral innate immunity (Figure 4). The A549-hACE2 cells exhibited an upregulation of the genes encoding for the CD80 molecule (CD80), FBJ murine osteosarcoma viral oncogene homolog (FOS), interferon alpha 1 and 2 (IFNA1 and IFNA2) and tumor necrosis factor (TNF) (2.83-, 3.07-, 2.91-, 8.98- and 3.57-fold regulation, respectively). In addition, the downregulation of interferon beta 1 (IFNB1, −7.52-fold regulation) and interleukin 12 alpha (IL12A, −17.2-fold regulation) was recorded. However, Dt treatment induced a down-regulation of interferon regulatory factor 7 (IRF7), mitogen-activated protein kinase 1 (MAP3K1), myxovirus (influenza virus) resistance 1, interferon-inducible protein p78 (mouse) (MX1), proline-serine-threonine phosphatase interacting protein 1 (PSTP1P1) and Toll-like receptors 7 and 9 (TLR7 and TLR9), (−2.2-, −2.16-, −5.44-, −7.37-, −13.66- and −2.74-fold regulation, respectively). Finally, the treatment with Dt led to a downregulation of Apolipoprotein B mRNA editing enzyme, catalytic polypeptide-like 3G (APOBEC3G) and chemokine (C-X-C motif) ligand 9 (CXCL9).

### 3.5. Anti-Inflammatory Response and Protein Expression

**Figure 5:**
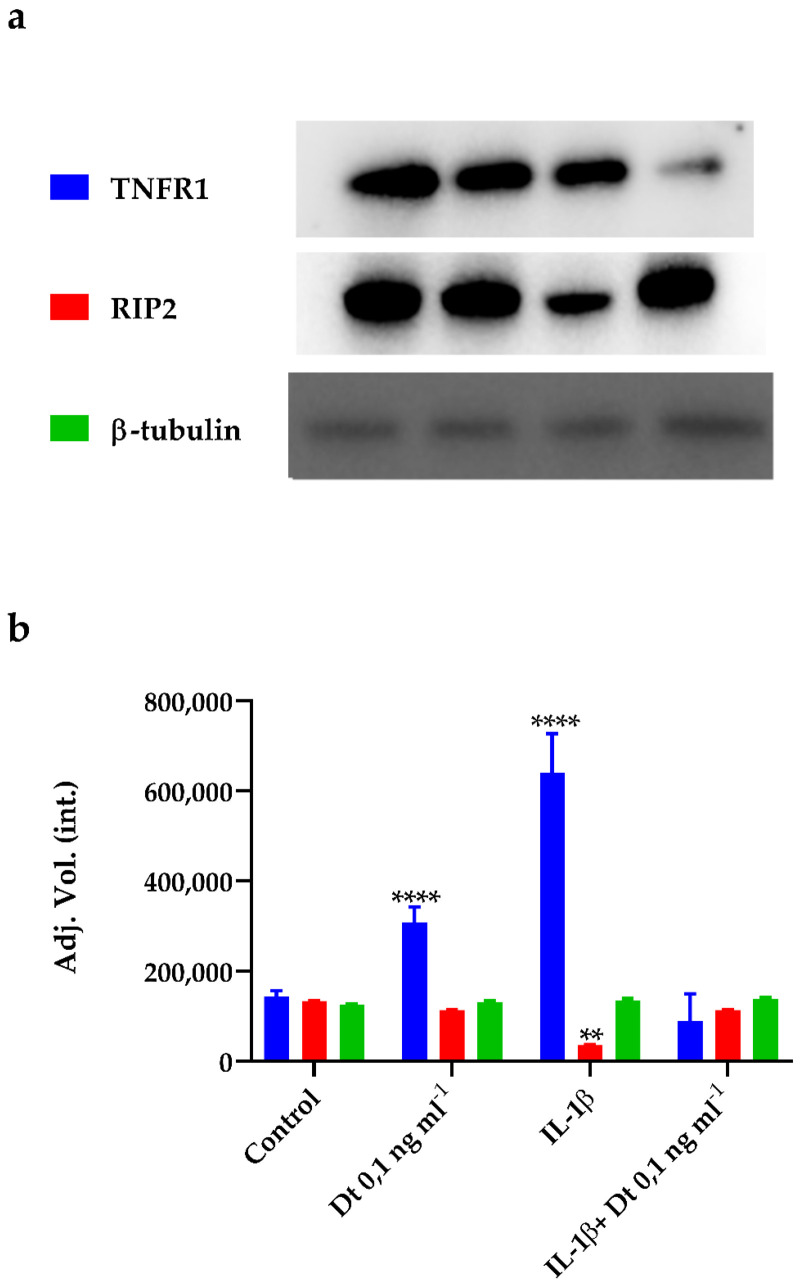
Expression of TNFR1, RIP2, and β-tubulin proteins in A549-hACE2 cells in different conditions, namely no treatment (Control), treatment for 48 h with Dt at 0.1 ng mL^−1^, 24 h stimulation with IL-1β (40 pg mL^−1^), and treatment for 48 h with Dt after a 24 h pre-stimulation with IL-1β (40 pg mL^−1^). Bands (**a**) obtained for the proteins were analysed by densitometric analysis to determine the absolute intensity (**b**). Values are expressed as mean ± SD. Asterisks indicate the statistically significant difference compared to the respective control (**** *p* ≤ 0.0001, ** *p* ≤ 0.01; Tukey’s test).

The expression levels of tumor necrosis factor receptor 1 (TNFR1) and receptor-interacting-serine/threonine-protein kinase 2 (RIP2) proteins were investigated in A549-hACE2 cells, treated or not with the pro-inflammatory interleukin 1β (IL-1β) [41] and with or without Dt addition. A549-hACE2 cells treated with Dt increased the TNFR1 protein expression, while no effect on RIP2 protein expression was observed (*p* < 0.0001 and *p* > 0.05, respectively; Figure 5). The exposure of A549-hACE2 cells to IL-1β determined a huge increase in TNFR1 protein expression and the decrease of RIP2 protein expression compared to the control (*p* < 0.0001 and *p* < 0.001, respectively; Figure 5). In this context, Dt decreased the TNFR1 protein expression to the control level (Figure 5; *p* < 0.001), and significantly enhanced RIP2 expression (Figure 5; *p* < 0.0001).

### 3.6. Cytokine Storm Inhibition

**Figure 6:**
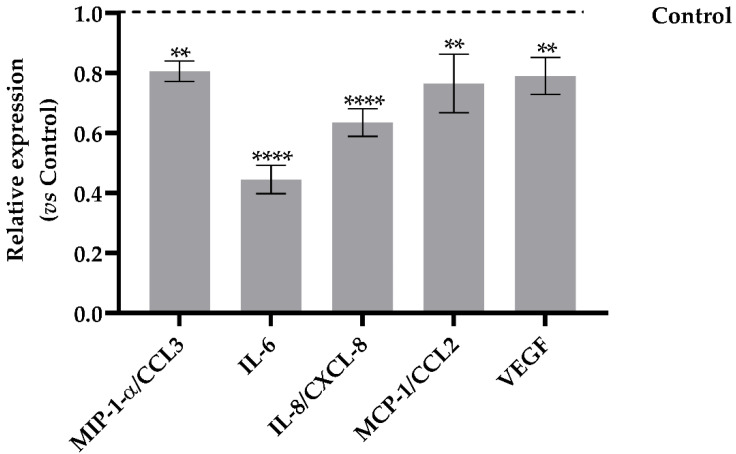
Human cytokines array analysis of the conditional medium from A549-hACE2 cells treated for 24 h with Dt at 0.1 ng mL^−1^, after a 24 h pre-stimulation with IL-1β (40 pg mL^−1^). Values are expressed as relative expression mean levels ± SD, obtained comparing the expression values to those of IL-1β control (**** *p* ≤ 0.0001, ** *p* ≤ 0.01; Sidak’s test).

Dt was able to act against the cytokine storm through the inhibition of the expression of the macrophage inflammatory proteins (MIP-1-α, Figure 6), as well of the cytokines interleukin 6 and 8 (IL-6 and IL-8), involved in the onset of the inflammatory storming that characterizes the innate immunity activation. In addition, a significant decrease of the relative expression of monocyte chemoattractant protein-1 (MCP-1, Figure 6) indicated a reduction in proinflammatory response involving immune cell recruitment. In parallel, the Dt-induced lowering of vascular endothelial growth factor (VEGF) production confirmed that inflammation and vasodilation were significantly inhibited (Figure 6).

## 4. Discussion

Coronavirus disease (COVID-19) is the etiologic factor for the pandemic novel severe acute respiratory syndrome-related coronavirus (SARS-CoV-2). The COVID-19 pandemic was similar to a “butterfly effect”, starting with the virus-mediated intracellular cytokine storm in lung cells and generating global economic and social disorders, together with a high mortality rate. This pandemic tells us that modern times’ biological infections might induce unthinkable chaos on Earth, thus critically needing the generation of healthy behaviors and products to enhance prevention against viral attack. The development and administration of vaccines by companies such as AstraZeneca/University of Oxford (UK), Institute Pasteur/Merck/Themis (France/USA/Austria), University of Hong Kong (China), CureVac (Germany), Moderna (USA), Inovio (USA), Clover Biopharmaceuticals (China), Novavax (USA), University of Queensland/CSL (Australia), and Sechenov University (Russia, Sputnik V) reached different phases, but no standard and specific treatment protocol has yet been developed. Vaccine efficacy depends on virus evolution and its mutation [42]. Prevention is worthwhile, both in the vision of National Health Systems towards pandemic shortages and extraordinary expenses, and in economical sustainability for patients. Therefore, it is crucial to invest in preventing actions to inhibit viral entry in the organisms. Targeting ACE2 is one of the promising approaches for preventing COVID-19 disease [43], since small compounds that are able to interact with the ACE2 receptor are likely to inhibit the attachment of SARS-CoV-2 onto the targeted human cells [17,44,45], or block the early interactions of SARS-CoV-2 with ACE2, as demonstrated for SSAA09E2 {N-[[4-(4-methyl-piperazin-1-yl)phenyl]methyl]-1,2-oxazole-5-carboxamide} or NAAE (N-(2-aminoethyl)-l-aziridine-ethanamine) [46]. In addition, chloroquine—a medication used for treating malaria—inhibits SARS-CoV-2 infection and the exacerbation of pneumonia, whereas side effects might be somehow important [47]. Seeking natural and eco-sustainable compounds able to significantly bind ACE2 is therefore essential. In this context, molecular docking to predict the capacity of molecules for binding ACE2, as well as *in vitro* tests using human cell lines and the SARS-CoV-2 spike protein, are tools allowing the bioprospecting of bioactive molecules potentially useful for health prevention [48,49,50,51,52]. 

Diatoxanthin is an exclusively aquatic microalgal xanthophyll with a (photo-)protective role [27,28,53]. Recent findings highlighted its outstanding bioactivity, with marked antioxidant, anti-inflammatory, and chemopreventive properties ([27,33] unpublished data). Interestingly, Dt can induce ferroptosis in cancer cell lines [33]. These results, together with the fact that Dt content in microalgae responds to external stresses (e.g., light, nutrients, iron, or viral attack) [31,32,50,54,55] lay the foundation to explore the potential of Dt as an inhibitor of the SARS-CoV-2-induced cytokine storm in lung cells. Results do strongly confirm this hypothesis, with a marked role of Dt in targeting the ACE2 receptor of tumor lung cells and inhibiting the cytokine storm following the contact between cells and the SARS-CoV-2 spike glycoprotein. Dt is eligible as a preventive/therapeutic agent against SARS-CoV-2 infection, cytokine storm consequences at the multiorgan level, and the long-term effects of infection due to the high levels of inflammatory markers in the blood [56]. A recent in vitro study highlighted that Dt displays antioxidant activity, and exerts an anti-inflammatory role [27]. Here, it is shown that Dt inhibits IL-6 release in lung cells exposed to the spike glycoprotein, and increases the IL-10 expression, providing an immunomodulatory signaling role. This result is strongly relevant since IL-6 increase is one of the biological responses responsible for severe respiratory injuries in patients affected by SARS-CoV-2 with dramatic involvement of coagulopathy and lung thrombosis after bilateral pneumonia [2]. The immunostimulant role of Dt involves the up-regulation of interferon alpha 1 and 2 (IFNA1 and IFNA2) and tumor necrosis factor (TNF), belonging to the intracellular innate system for the release of inflammatory mediators that induce specific differentiation in naïve monocytes [50]. The overexpression of interferon suggests an effective protection against ARDS, whereas recent studies demonstrated that patients with IFNAR1 deficiency were prone to develop severe forms of COVID-19 [57]. 

It is also noteworthy that Dt achieves its bioactive role in contrasting in vitro the first potential effects of SARS-CoV-2 at low concentration (0.1 ng mL^−1^), which is relevant and promising for potential therapy. A few cubic millimeters of diatom culture are enough to reach such a Dt content [31,32].

Moreover, it is important to consider that (Dt-containing) microalgae offer great opportunities as relevant eco-sustainable resources for the biotechnological panorama [58], being promising cell factories displaying fast growth capacity and being genetically and metabolically engineerable. Indeed, to be attractive—and not only regarding direct health benefit efficacy—the product needs to be sustainable, i.e., with a low productive cost, limited impact on the environment, and using renewable resources. In this framework, terrestrial plants would be limited/avoided as a resource for new compounds due to the environmental costs of terrestrial cultivation and the long time needed for growth (see the review of [53,59]). Microalgae, instead, sharing photosynthetic and microbial properties, are a promising eco-sustainable alternative [60,61,62,63,64,65]. Moreover, the microalgae content and diversity in bioactive compounds—for example, vitamins, carotenoids, or polyphenols [23,27,66,67,68]—make them highly attractive bio-factories [60,67,68,69]. 

The next step regarding industrial development involves the lowering of the productive cost through technological improvements, and/or the enhancement of the bioactive interests/quality of the biomass [70,71,72,73]. 

The results from this study, as well as the Dt-producing microalgae opportunities, pave the way for the further investigation, i.e., the in vivo assessment of the antiviral role of Dt, especially against the SARS-CoV-2 virus.

## Data Availability

All of the data is contained within the article and the Supplementary Materials.

## Supplementary Materials

The following supporting information can be downloaded at: https://www.mdpi.com/article/10.3390/antiox11081515/s1, Table S1: Fold regulation values of genes obtained from PCR-array gene expression analysis. Figure S1: Chemical structure of diatoxanthin (C_40_H_54_O_2_, CAS No. 31063-73-7.
