# Supplementary material for: The Microalgal Diatoxanthin Inflects the Cytokine Storm in SARS-CoV-2 Stimulated ACE2 Overexpressing Lung Cells
Source: Antioxidants (Basel). 2022 Aug 3;11(8):1515. doi: 10.3390/antiox11081515 (PMC9405469; doi:10.3390/antiox11081515)
Supplement: Supplementary file 1 [file antioxidants-11-01515-s001.zip › antioxidants-1836972-supplementary/Table S1_03.08.22.pdf]

Table S1. Fold regulation values of genes obtained from PCR-array gene expression analysis.

Gene Symbol	GenBank	Gene Name	Fold Regulation
AIM2	NM_004833	Absent in melanoma 2	1.81
APOBEC3G	NM_021822	Apolipoprotein B mRNA editing enzyme, catalytic polypeptide-like 3G	-6.01
ATG5	NM_004849	ATG5 autophagy related 5 homolog (*S. cerevisiae*)	-1.32
AZI2	NM_022461	5-azacytidine induced 2	-1.43
CARD9	NM_052813	Caspase recruitment domain family, member 9	-1.24
CASP1	NM_033292	Caspase 1, apoptosis-related cysteine peptidase (interleukin 1, beta, convertase)	-1.16
CASP10	NM_001230	Caspase 10, apoptosis-related cysteine peptidase	-1.24
CASP8	NM_001228	Caspase 8, apoptosis-related cysteine peptidase	-1.78
CCL3	NM_002983	Chemokine (C-C motif) ligand 3	1.15
CCL5	NM_002985	Chemokine (C-C motif) ligand 5	-1.20
CD40	NM_001250	CD40 molecule, TNF receptor superfamily member 5	1.18
CD80	NM_005191	CD 80 molecule	2.83
CD86	NM_006889	CD86 molecule	-1.24
CHUK	NM_001278	Conserved helix-loop-helix ubiquitous kinase	-1.16
CTSB	NM_001908	Cathepsin B	-1.44
CTSL1	NM_001912	Cathepsin L1	-1.29
CTSS	NM_004079	Cathepsin S	-1.27
CXCL10	NM_001565	Chemokine (C-X-C motif) ligand 10	1.03
CXCL11	NM_005409	Chemokine (C-X-C motif) ligand 11	-1.04
CXCL9	NM_002416	Chemokine (C-X-C motif) ligand 9	-2.74
CYLD	NM_015247	Cylindromatosis (turban tumor syndrome)	-1.25
DAK	NM_015533	Dihydroxyacetone kinase 2 homolog (S. cerevisiae)	1.05
DDX3X	NM_001356	DEAD (Asp-Glu-Ala-Asp) box polypeptide 3, X-linked	-1.18
DDX58	NM_014314	DEAD (Asp-Glu-Ala-Asp) box polypeptide 58	-1.39
DHX58	NM_024119	DEXH (Asp-Glu-X-His) box polypeptide 58	-1.61
FADD	NM_003824	Fas (TNFRSF6)-associated via death domain	1.46
FOS	NM_005252	FBJ murine osteosarcoma viral oncogene homolog	3.07
HSP90AA1	NM_001017963	Heat shock protein 90kDa alpha (cytosolic), class A member 1	-1.11
IFIH1	NM_022168	Interferon induced with helicase C domain 1	-1.13
IFNA1	NM_024013	Interferon, alpha 1	2.91
IFNA2	NM_000605	Interferon, alpha 2	1.98
IFNAR1	NM_000629	Interferon (alpha, beta and omega) receptor 1	1.01
IFNB1	NM_002176	Interferon, beta 1, fibroblast	-7.52
IKBKB	NM_001556	Inhibitor of kappa light polypeptide gene enhancer in B-cells, kinase beta	-1.47
IL12A	NM_000882	Interleukin 12A (natural killer cell stimulatory factor 2, cytotoxic lymphocyte maturation factor 1, p35)	-17.20
IL12B	NM_002187	Interleukin 12B (natural killer cell stimulatory factor 2, cytotoxic lymphocyte maturation factor 1, p40)	1.18
IL15	NM_000585	Interleukin 15	1.39
IL18	NM_001562	Interleukin 18 (interferon-gamma-inducing factor)	-1.41
IL1B	NM_000576	Interleukin 1, beta	-1.04
IL6	NM_000600	Interleukin 6 (interferon, beta 2)	-1.13
IL8	NM_000584	Interleukin 8	1.50
IRAK1	NM_001569	Interleukin-1 receptor associated kinase 1	-1.25
IRF3	NM_001571	Interferon regulatory factor 3	-1.40

IRF5	NM_001098629	Interferon regulatory factor 5	-1.16
IRF7	NM_001572	Interferon regulatory factor 7	-2.20
ISG15	NM_005101	ISG15 ubiquitin-like modifier	1.07
JUN	NM_002228	Jun proto-oncogene	1.39
MAP2K1	NM_002755	Mitogen-activated protein kinase kinase 1	-1.02
MAP2K3	NM_002756	Mitogen-activated protein kinase kinase 3	-1.13
MAP3K1	NM_005921	Mitogen-activated protein kinase kinase kinase 1	-2.16
MAP3K7	NM_003188	Mitogen-activated protein kinase kinase kinase	-1.22
MAPK1	NM_002745	Mitogen-activated protein kinase 1	-1.38
MAPK14	NM_001315	Mitogen-activated protein kinase 14	-1.82
MAPK3	NM_002746	Mitogen-activated protein kinase 3	1.25
MAPK8	NM_002750	Mitogen-activated protein kinase 8	-1.37
MAVS	NM_020746	Mitochondrial antiviral signalling protein	1.26
MEFV	NM_000243	Mediterranean fever	-1.24
MX1	NM_002462	Myxovirus (influenza virus) resistance 1, interferon-inducible protein p78 (mouse)	-5.44
MYD88	NM_002468	Myeloid differentiation primary response gene (88)	-1.09
NFKB1	NM_003998	Nuclear factor of kappa light polypeptide gene enhancer in B-cells 1	1.04
NFKBIA	NM_020529	Nuclear factor of kappa light polypeptide gene enhancer in B-cells inhibitor, alpha	1.00
NLRP3	NM_183395	NLR family, pyrin domain containing 3	-1.24
NODE2	NM_022162	Nucleotide-binding oligomerization domain containing 2	-1.36
OAS2	NM_002535	2′5′-oligoadenylate synthetase 2, 69/71kDa	-1.29
PIN1	NM_006221	Peptidylprolyl cis/trans isomerase, NIMA-interacting 1	-1.01
PSTPIP1	NM_003978	Proline-serine-threonine phosphatase interacting protein 1	-7.37
PYCARD	NM_013258	PYD and CARD domain containing	-1.98
PYDC1	NM_152901	PYD (pyrin domain) containing 1	-1.32
RELA	NM_021975	V-rel reticuloendotheliosis viral oncogene homolog A (avian)	-1.08
RIPK1	NM_003804	Receptor (TNFRSF)-interacting serine-threonine kinase 1	1.06
SPP1	NM_000582	Secreted phosphoprotein 1	-1.38
STAT1	NM_007315	Signal transducer and activator of transcription 1, 91 kDa	-1.38
SUGT1	NM_006704	SGT1, suppressor of G2 allele SKP1 (*S. cerevisiae*)	-1.04
TBK1	NM_013254	TANK-binding kinase 1	-1.23
TICAM1	NM_182919	Toll-like receptor adaptor molecule 1	-1.21
TLR3	NM_003265	Toll-like receptor 3	-1.50
TLR7	NM_016562	Toll-like receptor 7	-13.66
TLR8	NM_138636	Toll-like receptor 8	-1.24
TLR9	NM_017442	Toll-like receptor 9	-2.74
TNF	NM_000594	Tumor necrosis factor	3.57
TRADD	NM_003789	TNFRSF1A-associated via death domain	1.40
TRAF3	NM_003300	TNF receptor-associated factor 3	-1.21
TRAF6	NM_004620	TNF receptor-associated factor 6	1.17
TRIM25	NM_005082	Tripartite motif containing 25	-1.10
