# Supplementary figures and images for: The Microalgal Diatoxanthin Inflects the Cytokine Storm in SARS-CoV-2 Stimulated ACE2 Overexpressing Lung Cells
Source: Antioxidants (Basel). 2022 Aug 3;11(8):1515. doi: 10.3390/antiox11081515 (PMC9405469; doi:10.3390/antiox11081515)

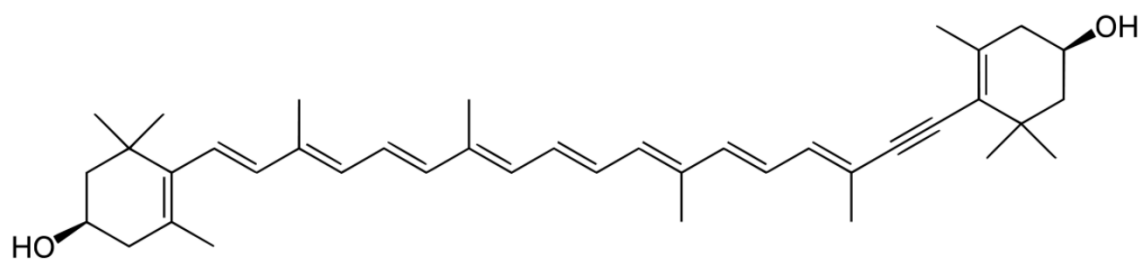

**Figure S1** Chemical structure of diatoxanthin ( $C_{40}H_{54}O_2$ , CAS No. 31063-73-7).

Supplement: Supplementary file 1 [file antioxidants-11-01515-s001.zip › antioxidants-1836972-supplementary/Figure S1_03.08.22.pdf]
